# Streamlining trigger delay estimation for T1 mapping

**DOI:** 10.1186/1532-429X-18-S1-T11

**Published:** 2016-01-27

**Authors:** George L Mathew, Vassilis Vassiliou, Ee Ling Heng, Gillian C Smith, Sri Anita, Nishad Unnikrishnan, Francisco Alpendurada, Dudley J Pennell, Peter Gatehouse, Karen Symmonds, Sanjay Prasad

**Affiliations:** 1 CMR, Royal Brompton Hospital, London, United Kingdom; 2 National Heart and Lung institute, Imperial College London, London, United Kingdom; 3 Medicine, Imperial College London, London, United Kingdom

## Background

T1 mapping in end diastole requires correct identification of the diastolic pause, and an appropriate input of the trigger delay time (TD) into acquisition parameters to ensure parametric mapping during the true diastolic pause for minimal motion-related blurring of myocardium. This diascan can be derived for individual patients from either the 4 chamber or the short axis stack. However, this additional step adds complexity and time to the scan and potentially introduces error. We investigated whether the TD could be estimated from the heart rate (HR) to simplify MOLLI acquisitions.

## Methods

Patients who had a clinical scan were randomly selected retrospectively and grouped by their mean heart rate (n = 70, 10 patients per stepwise10HB group) as shown in table 1. These patients had a mixture of conditions including valvular disease, coronary disease, cardiomyopathy and normal scans. Cardiac diastasis duration and TD were measured from cine images as per the formula TD=((time at beginning of diastolic stasis)+(time at end of diastolic stasis)/2) for each patient. Assuming a linear relationship a line of best fit was calculated for the 70 measurements. From the line-fit, TD could be estimated for any prospective scan on the basis of HR. The calculated values were validated against a separate cohort of retrospective patients (n = 50, 10 patients per group 2-6). Then prospectively 10 volunteers (age 29 yrs, 5 male) underwent native T1 mapping (Siemens investigational prototype WIP 448B) using the calculated TD and the estimated TD to allow comparison. A low or high resolution version of the sequence was used if HR was < or >90 bpm. Each had basal and mid level T1 mapping repeated twice, and using the average of the two levels a total of 10 basal and 10 mid level values for the whole cohort were available for comparison.

## Results

**Figure 1:**
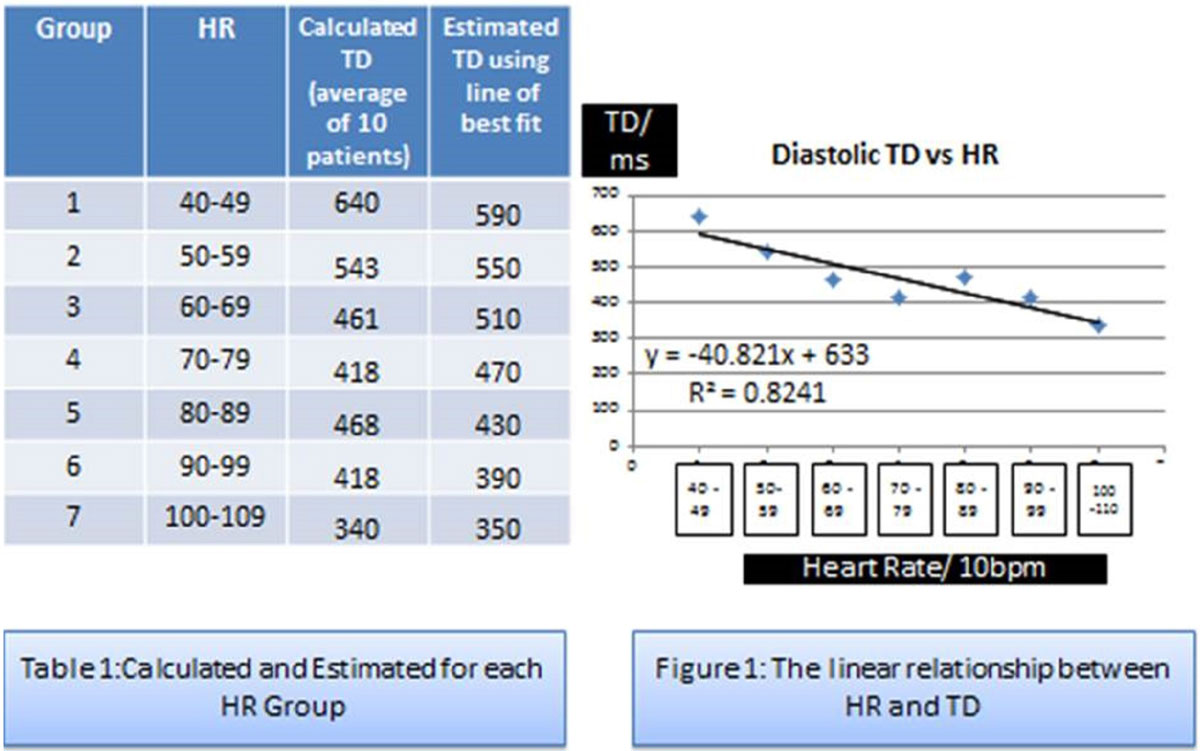
**left showing calculated and estimated TD for each HR group, right showing the lines relationship between HR and TD**.

There was a strong linear relationship between each HR group and calculated TD, Estimated TD = -40.82 × HR +633, R2 = 0.82 (Figure 1) suggesting that it is possible to estimate correctly the TD on the basis of the HR (Figure 1). Applying this equation to the n = 50 retrospective validation cohort showed good correlation (R2 = 0.84) between the estimated and the calculated TDs. For the prospective volunteers, the calculated and estimated TD had modest correlation (ICC 0.43; R2 = 0.65). However, the T1 maps from the calculated and estimated TD showed strong correlation (ICC = 0.82, R2 = 0.76, Bland Altman plot figure 3).

**Figure 2:**
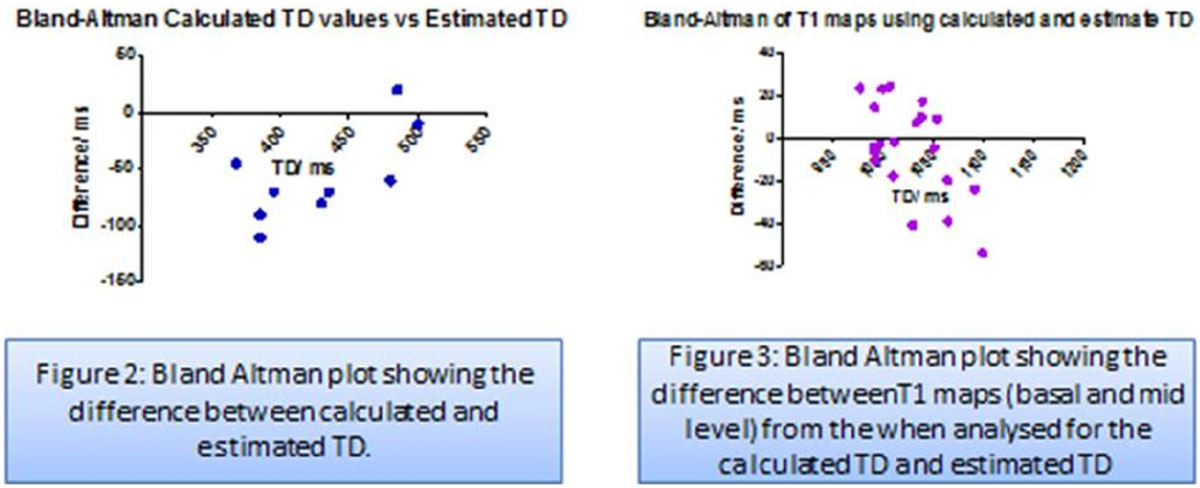
**left showing the Bland Altman plot showing the difference between calculated and estimated TD, right, showing the Bland Altman plot of the difference between T1 maps using the calculated and estimated TD**.

## Conclusions

This work suggests that the estimated TD may provide a suitable alternative to manual calculations of TD for patients scheduled for T1 mapping using an 11HB MOLLI. Larger studies might allow a more accurate estimation of the TD particularly if more factors are modeled, rather than HR alone in multiple pathologies. However, at present pre-patient optimization of TD from their cine images remains more accurate and should be considered as the default.

